# The effect of inlet flow profile and nozzle diameter on drug delivery to the maxillary sinus

**DOI:** 10.1007/s10237-022-01563-8

**Published:** 2022-02-08

**Authors:** Oveis Pourmehran, Benjamin Cazzolato, Zhao Tian, Maziar Arjomandi

**Affiliations:** School of Mechanical Engineering, The University of Adelaide, North Terrace, Adelaide, SA 5005 Australia

**Keywords:** CFD simulation, Targeted drug delivery, Maxillary sinus

## Abstract

In this paper, the effect of the turbulence and swirling of the inlet flow and the diameter of the nozzle on the flow characteristics and the particles' transport/deposition patterns in a realistic combination of the nasal cavity (NC) and the maxillary sinus (MS) were examined. A computational fluid dynamics (CFD) model was developed in ANSYS® Fluent using a hybrid Reynolds averaged Navier–Stokes–large-eddy simulation algorithm. For the validation of the CFD model, the pressure distribution in the NC was compared with the experimental data available in the literature. An Eulerian–Lagrangian approach was employed for the prediction of the particle trajectories using a discrete phase model. Different inlet flow conditions were investigated, with turbulence intensities of 0.15 and 0.3, and swirl numbers of 0.6 and 0.9 applied to the inlet flow at a flow rate of 7 L/min. Monodispersed particles with a diameter of 5 µm were released into the nostril for various nozzle diameters. The results demonstrate that the nasal valve plays a key role in nasal resistance, which damps the turbulence and swirl intensities of the inlet flow. Moreover, it was found that the effect of turbulence at the inlet of the NC on drug delivery to the MS is negligible. It was also demonstrated that increasing the flow swirl at the inlet and decreasing the nozzle diameter improves the total particle deposition more than threefold due to the generation of the centrifugal force, which acts on the particles in the nostril and vestibule. The results also suggest that the drug delivery efficiency to the MS can be increased by using a swirling flow with a moderate swirl number of 0.6. It was found that decreasing the nozzle diameter can increase drug delivery to the proximity of the ostium in the middle meatus by more than 45%, which subsequently increases the drug delivery to the MS. The results can help engineers design a nebulizer to improve the efficiency of drug delivery to the maxillary sinuses.

## Introduction

The nasal cavity is an important organ in the human body with critical functions such as humidification and heating of inhaled air and filtration of pollutants from inhaled air (Drettner et al. 1977; Keck et al. 2000). The surface of the nasal cavity is mostly lined with mucosa. The mucosa is highly vascularized, such that the amount of blood flow to the surface of the NC is higher than that of blood flow to the brain (Mygind et al. 1978). The mucosal surface of NC, which is highly vascularized, provides an attractive route for the treatment of sinus-related diseases, such as chronic rhinosinusitis (CRS) (Bell et al. 2012; Rissler et al. 2012; Wichers et al. 2006).

**Fig. 1:**
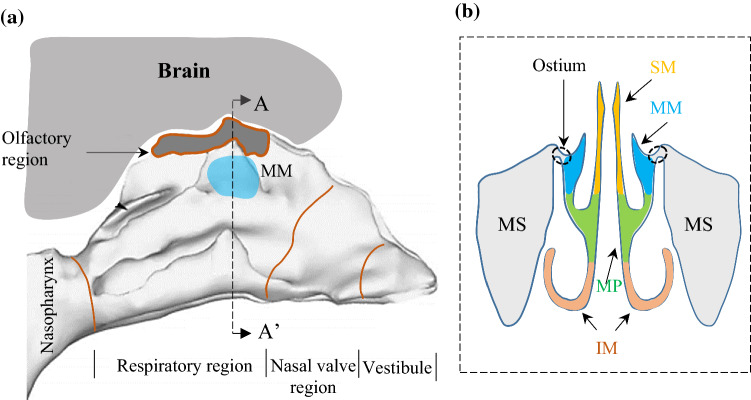
**a** An overview of the NC, representing the vestibule, nasal valve region, the respiratory region, olfactory region, middle meatus (MM) and nasopharynx, adapted from Shi et al. (2007) with permission from Elsevier; b section A-A’ representing the maxillary sinuses (MS), ostium, and superior meatus (SM), middle meatus (MM), main passage, (MP), inferior meatus (IM), adapted from Xi et al. (2017) with permission from Elsevier

Human NC is constructed by convoluted airways. The maxillary sinus (MS) is located on the lateral side of the NC, where it is difficult to access (see Fig. 1b). The only opening that can accommodate the delivery of medication to the MS is the ostium, which is located in the middle meatus (MM). The locations of MS contribute to some of the challenges for achieving efficient drug delivery to those regions because most inhaled medications either deposit in the anterior region or pass through the inferior meatus and the main passage of the NC, which are far from the target site, the middle meatus and the MS (see Fig. 1b) (Bahadur et al. 2012; Xi et al. 2015).

For drug delivery to the MS, two components should be considered: the delivery of the drug to the MM where the ostium is located; and the delivery of the drug particles from the MM to the MS (see Fig. 1b). The latter component has already been investigated in previous studies, which showed that nasal sprays and standard nebulisers (either jet or mesh nebulisers) are not able to efficiently deliver aerosolised medications to the MS (Berger et al. 2007; Hilton et al. 2008b; Möller et al. 2014; Wofford et al. 2015). Instead, an active drug delivery technique is required to drive the drug particles from the MM to the MS. Studies have shown that the most efficient, noninvasive, and low-cost method is acoustic drug delivery (ADD) (Navarro et al. 2019).

In ADD, an acoustic wave is superimposed on the aerosol entering the NC through the nostril. The acoustic wave leads to an oscillation of airflow in the NC, which causes a pressure difference between the NC and MS, resulting in enhanced flow exchange between the NC and MS (Leclerc et al. 2015). Momentum exchange between the MS and NC is the primary requirement for the delivery of the drug particles to the MS through the ostium. The performance of ADD for MS highly depends on the aero-acoustic parameters such as input frequency and sound pressure level (SPL) (Durand et al. 2011). Several studies have investigated the aerosol deposition in the MS using ADD with different input frequencies and SPL, which demonstrated a three- to fourfold increase in the aerosol deposition in the MS compared with conventional nebulisation without acoustics (Durand et al. 2011; Leclerc et al. 2014; Maniscalco et al. 2013, 2006).

**Fig. 2:**
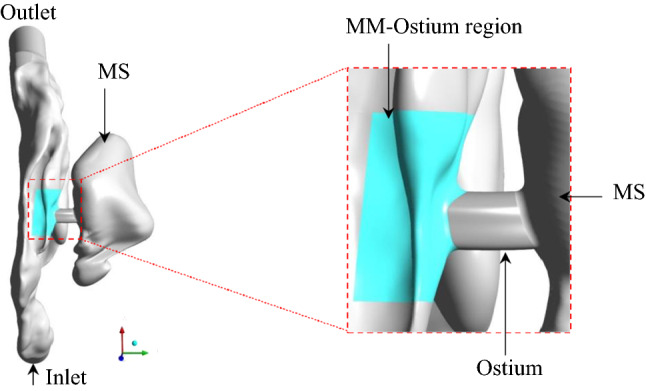
The top view of the MC-MS model and the MM-Ostium region marked in cyan

Recently, Pourmehran et al. (2021) demonstrated that drug deposition in MS can be increased 45-fold using a well-designed acoustic wave. They also showed that the particle deposition in the MS changes with the mass flow rate of particles entering the nostril, even though an optimal acoustic wave was applied to the nostril. This implies that the efficiency of ADD depends not only on the aero-acoustic parameters but also on the retention and concentration (number) of particles passing through the MM-ostium region because, by changing the nebulisation flow rate, the number of particles passing through the MM-ostium region is likely to vary. MM-Ostium refers to a region in the MM where the ostium connects the MM to the MS as illustrated in Fig. 2. It is hypothesised that an increase in the concentration of particles passing through the MM can increase the ADD efficiency proportionally, which is related to the former component of drug delivery to the MS, as described above. This issue is the main motivation of the current study, which led us to investigate the effect of different controllable parameters on the retention and concentration of particles in the MM-ostium region. To realise these parameters, a comprehensive literature review on available drug delivery methods and were carried and a brief summary are presented in the following paragraphs. It should be noted that in this study, the term particle refers to the droplet generated by a nebuliser.

Various methods and devices are available for nasal drug delivery such as nasal sprays and nebulisers. Nasal sprays produce particles with diameters in the range of 50–100 μm, which almost entirely deposit in the anterior region of the nasal cavity, and are not capable of delivering the particles to the meatuses and posterior regions of the NC, which limits their application for delivery of medication to the MS (Shi et al. 2006, 2007; Tong et al. 2016; Xi et al. 2008). The application of nebulisers, however, has been shown to be an efficient method to overcome the challenges related to the limitation of delivery of particles to the posterior region of the NC and the meatuses (Laube, 2007). Nebulisers produce fine particles in a range of 1–30 μm, which enables particle to reach the posterior region (Hilton et al. 2008a; Wofford et al. 2015). However, particle transport in the middle meatus is considerably lower than that of the inferior meatus when a nebuliser is used (Zhao et al. 2004). On the other hand, different studies showed that particle deposition in respiratory airways is impacted by inhaled flow patterns. Lin et al. (2012) examined the effect of nebuliser types and aerosol face masks on the efficiency of drug delivery to the NC. They reported that the design of an aerosol mask affects the dose of the inhaled aerosolised drug for different types of nebulisers. Using jet and mesh nebulisers, Ari et al. (2015) investigated the impact of airflow rates on particle deposition in the lung. They demonstrated that the drug delivery efficiency using a mesh nebuliser was higher than that using a jet nebuliser. They also found that the efficiency of drug delivery to the lung through the valve mask is higher than that of a standard open aerosol mask. Hence, it can be inferred that the inlet airflow features, and aerosol distribution applied at the nostril can have an impact on aerosol deposition and transport in different regions of the NC.

To address the existing gaps (i.e. the impact of flow features on drug delivery to MM-Ostium region), this study instigates the effect of inlet flow turbulence intensity and swirl number, and the nozzle diameter in the nostril on the drug delivery to the MM-Ostium. To do so, various turbulent intensities and swirl numbers were applied at the nostril, and their impact on the flow structure in the NC-MS combination, as well as on the transport/deposition patterns of the particle in the MM-Ostium region, was quantified. The effect of the nozzle diameter (the diameter of injection of particles at the inlet) on the aerosol deposition and transport pattern was also studied using computational fluid dynamics (CFD) modelling. CFD is an advantageous noninvasive tool to study the airflow behaviour and particle transport pattern in the upper and lower airways (Inthavong et al. 2021; Islam et al. 2021; Rahman et al. 2021; Salati et al. 2021; Singh et al. 2020).

## Methods

### Nasal cavity geometry

**Fig. 3:**
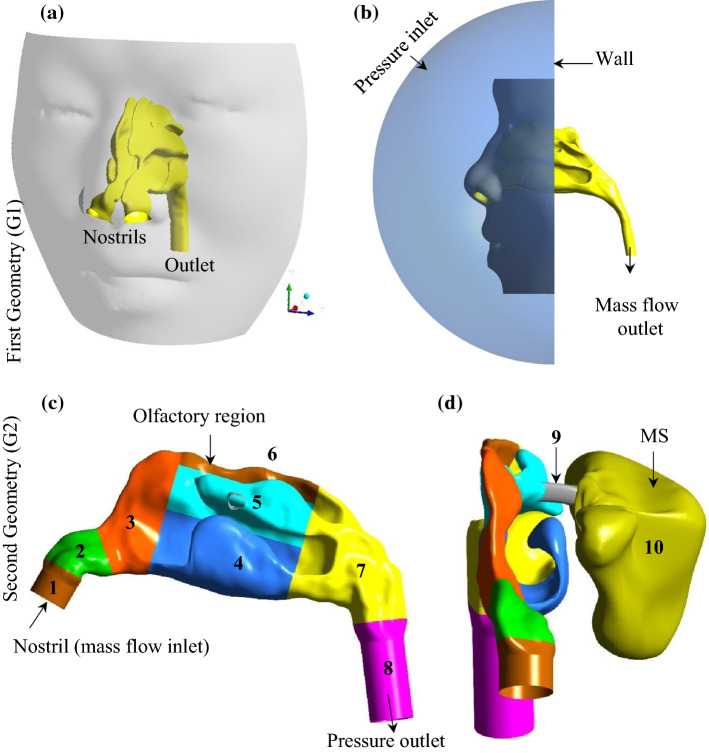
**a** Sagittal view and **b** isometric view of the first geometry (G1) (the STL model of the geometry was adapted from Van Strien et al. (2021) with permission; c sagittal and d frontal view of the second geometry (G2). The NC part of G2 was extracted from a CAD model of a realistic human respiratory system adapted from Tian et al. (2008), and the MS part of G2 was generated based on a realistic nose geometry adapted from Kumar et al. (2016). G2 was segmented into 10 zones. Each zone is defined by a colour and a number between 1 and 10

In this study, two different nose geometries were used. The first geometry (G1), which excludes the paranasal sinuses, was used for validation of the CFD model. It was necessary to use G1 since the experimental data for the validation of the CFD model were available for G1 in a recently published article by Van Strien et al. (2021). The second geometry (G2), which includes the MS, was used to conduct parametric studies. The STL (stereolithography) file of G1 was adapted from Van Strien et al. (2021). The STL file was imported into ANSYS® SpaceClaim to convert the point cloud to a CAD (computer-aided design) format using the Shrink Wrap technique to improve the quality of the model by smoothening the surfaces. An external facial feature (Fig. 3a) in the shape of a hemisphere (Fig. 3b) was also added to the computational domain to ensure simulation of a realistic condition for inhalation through the nostril (Van Strien et al. 2021). After ensuring the validity of the CFD model by comparing the G1 inputs with the experimental data, the geometry G2 was used to perform parametric studies to investigate the effect of the inlet flow parameters and nozzle diameter on the flow features and particle transport in the NC. To measure particle deposition in different parts of the NC-MS combination, G2 was segmented into 10 zones (see Fig. 3c, d).

### Case studies

In this study, two different types of inlet flow were considered: turbulent (2 cases) and laminar (3 cases). For turbulent cases, two different turbulence intensities of ${\mathrm{TI}}_{\mathrm{in}}$ = 0.15 and ${\mathrm{TI}}_{\mathrm{in}}$ = 0.3 were considered. Turbulence intensity is defined as the ratio of the standard deviation of the fluctuating flow velocity to the mean flow velocity (Kimura 2016). The turbulent cases were produced by applying artificial turbulence to the laminar inlet flow with a Reynolds number of ($\mathrm{Re}=1023$). The laminar flow cases include a non-swirling inlet flow (${S}_{n}$ = 0) and two swirling inlet flows (${S}_{n}$ = 0.6 and ${S}_{n}$ = 0.9). The term ${S}_{n}$ refers to the swirl number, which quantifies the swirl intensity of the swirling flow defined as the ratio of the tangential momentum flux to the axial momentum flux at the inlet boundary (Hreiz et al. 2011) given by:

1 $$S_{n} = \frac{{\smallint ru_{t} u_{a} {\text{dA}}}}{{R\smallint ru_{a}^{2} {\text{dA}}}}$$

where $r$, ${u}_{t}$, ${u}_{a}$, and $R$ are the radial coordinate, tangential velocity, axial velocity, and the radius of the inlet boundary, respectively.

To take into account both the instantaneous number of particles and their residence time in the MM-Ostium region, the particles’ retention criterion was defined by:

2 $$N_{p}^{*} = \smallint N_{p} {\text{dt}}$$

where ${N}_{p}$ is the instantaneous number of particles in the target region and $t$ is the time. ${N}_{p}^{*}$ is the particles’ retention criterion calculated by the integration of the number of particles in a target region with respect to the particle time in a specific time frame. The time commences when the particles are released into the inlet.

**Fig. 4:**
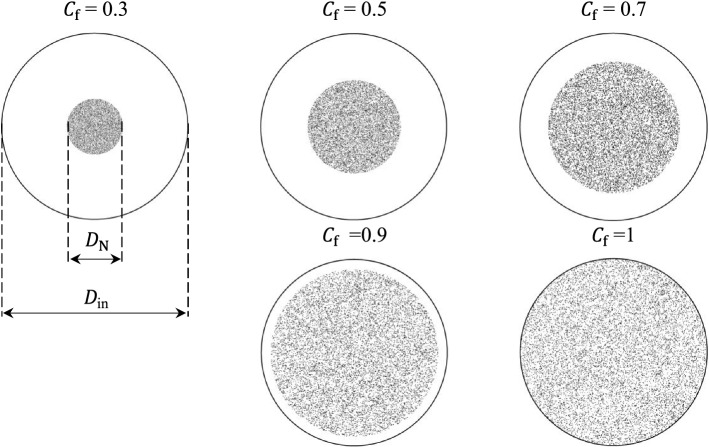
A snapshot of monodispersed particle distribution in the inlet for different fullness coefficient (${C}_{f}$). ${D}_{N}$: nozzle diameter (the diameter of injection of the particles), ${D}_{\mathrm{in}}$: inlet diameter

The effect of how particles are released in the inlet on drug delivery to the MM-Ostium region was also studied by considering five different fullness coefficients (${C}_{f}$), defined as the ratio of nozzle diameter to the inlet (nostril) diameter given by

3 $$C_{f} = \frac{{D_{N} }}{{D_{{{\text{in}}}} }}$$

where ${D}_{N}$ is the diameter of the nozzle for injecting the particles at the inlet and ${D}_{\mathrm{in}}$ is the diameter of the inlet boundary. Figure 4 illustrates the particle distribution for different ${C}_{f}$ in the inlet boundary. The particles were injected into the inlet with a random position distribution.

### Governing equations and boundary conditions

#### Fluid phase modelling

ANSYS® Fluent (2020) R1 was used to simulate the airflow and particle transport/deposition patterns in a realistic NC-MS model. The working fluid was considered as air, which was assumed to be Newtonian and incompressible. To investigate the particle deposition and transport pattern, as well as to isolate the outcomes independently from the oscillation variables, a constant inhalation airflow was used instead of a periodic inhalation flow. To ensure the validity of the use of a constant inhalation flow, the impact of cyclic inhalation on the airflow field was evaluated through the Womersley number (*W*) (Inthavong et al. 2008). *W* is a non-dimensional number indicating the effect of oscillatory flow on the flow behaviour in an internal flow, such as airflow in the NC and blood flow in the blood vessels (Loudon et al. 1998). The fluid flow behaves similarly to a quasi-steady state when *W* < 1, but the flow behaviour is far from a quasi-steady state when *W* > 1 [46]. The *W* is defined as:

4 $$W = \frac{L}{2}\sqrt {\left( {\frac{{\rho_{f} f}}{\mu }} \right)}$$

where $L$ is the characteristic length, which is identified as the distance between the two walls of the NC (Inthavong et al. 2008), ${\rho }_{f}$ is the fluid density, $\mu$ is the dynamic viscosity of the fluid, and $f$ is the frequency of breathing, which is approximately 15 cycles per minute.

In this study, the characteristic length varies between 1 and 10 mm for the NC, which results in 0.065 < *W* < 0.65. Hence, the assumption of quasi-steady state used in this study is valid for airflow modelling. It is worth mentioning that the quasi-steady state does not refer to a constant flow field over time, but it implies that the instantaneous flow rate is characterised by the instantaneous pressure gradient (Loudon et al. 1998). Regarding the effect of periodic flow on particle deposition, Haubermann et al. (2002) demonstrated that the impact of periodic inhalation-exhalation flow on particle deposition in NC is similar to the pattern of particle deposition under constant inhalation when the flow rate is *Q* = 10 L/min or less (Tian et al. 2008). Given that the flow rate used in the current study is *Q* = 7 L/min, the assumption of a constant inhalation flow rate is valid for the particle deposition in the NC-MS model (G2).

For cases with turbulent inlet flow, Stress-Blended Eddy Simulation (SBES), which is a Hybrid RANS-LES (RANS: Reynolds-averaged Navier–Stokes, LES: Large-Eddy Simulation) turbulence model, was used. In the SBES model, LES is used to resolve the flow field beyond the near-wall region, while the RANS model is applied to the near-wall region, which overcomes the need for very small grids in the near-wall region in turbulent modelling of LES (Van Strien et al. 2021). The equation of mass conservation in an incompressible flow is given by:

5 $$\frac{{\partial \overline{u}_{i} }}{{\partial x_{i} }} = S_{m} ,$$

where $\overline{u }$ is the flow velocity and ${S}_{m}$ is a source term originating from the mass added to or subtracted from the fluid due to different phenomena, such as vaporisation of the droplets. In this study, ${S}_{m}$ is zero. The equation describing the conservation for momentum in an incompressible flow is given by:

6 $$\frac{\partial }{\partial t}\left( {\rho_{f} \overline{u}_{i} } \right) + \frac{\partial }{{\partial x_{j} }}\left( {\rho_{f} \overline{u}_{i} \overline{u}_{j} } \right) = \frac{\partial }{{\partial x_{j} }}\left( {\rho_{f} \sigma_{{{\text{ij}}}} } \right) - \frac{{\partial \overline{p}}}{{\partial x_{i} }} + \frac{{\partial \tau_{{{\text{ij}}}} }}{{\partial x_{j} }} ,$$

where ${\rho }_{f}$, ${\overline{u} }_{i}$, $p$, ${\sigma }_{\mathrm{ij}}$, and ${\tau }_{\mathrm{ij}}$ are the density of the fluid, vector of the flow velocity, pressure field, stress tensor due to molecular viscosity, and the turbulence stress tensor ${\tau }_{\mathrm{ij}}={\rho }_{f}\left(\overline{{u }_{i}{u}_{j}}-{\overline{u} }_{i}{\overline{u} }_{j}\right)$, respectively (Fluent Theory Guide 2020). The overbar on the velocity and pressure scalars (${\overline{u} }_{i}$ and $\overline{p }$) indicates the Reynolds-averaging and spatial-temporal filtering operations in the RANS and LES models, respectively (Van Strien et al. 2021). In the SBES turbulence model, a blending function is used to blend the turbulence stress tensor, ${\tau }_{\mathrm{ij}}$, between the Reynolds and subgrid-scale stress tensors for the RANS and LES formulations, respectively (Fluent Theory Guide 2020). The blending function is given by:

7 $$\tau_{{{\text{ij}}}} = f_{s} \tau_{{\text{ij, RANS}}} + \left( {1 - f_{s} } \right)\tau_{{\text{ij, LES}}}$$

where ${f}_{s}$ is the shielding function with values between zero and unity, ${\tau }_{\mathrm{ij}, \mathrm{RANS}}$ is the Reynolds stress tensor, and ${\tau }_{\mathrm{ij}, \mathrm{LES}}$ is the subgrid-scale stress tensor.

To implement the SBES turbulent model in ANSYS® Fluent, the *k-ω* SST (Shear Stress Transport) model was utilised because the SBES model is embedded in the *k-ω* SST model. Also, a WALE (Wall-Adapting local Eddy-Viscosity) model was used for the subgrid-scale component. A pressure-velocity coupled solver was used to solve the governing equations. A bounded second-order implicit scheme was used for the transient formulation, the second-order scheme was employed for the discretisation of the pressure, and the second-order bounded schemes were used for the convective terms. The least-squares cell-based method was used to calculate the gradients.

#### Particle phase modelling

To predict the transport of the particle phase in a fluid, two different approaches can be applied: Euler–Euler and Euler–Lagrange approaches. In the Euler–Lagrange approach, the fluid phase is modelled as a continuum phase, while the particle phase is treated as a discretised phase, which is tracked in the Lagrangian reference frame (Adamczyk et al. 2014). The Euler–Lagrange approach was used in this study because the physical description and details of the particle transport and deposition patterns can be determined. To predict the particle transport and deposition patterns in the NC-MS combination, a discrete phase model (DPM) was used, where the trajectory of the particle phase can be predicted by integrating the force balance on the particle. The force balance equation on a particle is defined by:

8 $$m_{p} \frac{{{\text{d}}\vec{u}_{p} }}{{{\text{dt}}}} = \vec{F}_{g} + \vec{F}_{D} ,$$

where ${m}_{p}$, ${u}_{p}$, ${\overrightarrow{F}}_{g}$, and ${\overrightarrow{F}}_{D}$ are the particle mass, velocity, gravitational force, and drag force, respectively. Other forces such as virtual mass, pressure gradient, Basset, Faxen, Saffman lift, and Brownian forces were neglected. The virtual mass, Basset, and pressure-gradient forces are considered when the density of the fluid is much greater than the density of the particles (Bassett 1888; Kolev 2011; Maul 2019). In this study, the density of the fluid is much smaller than that of particles (${\rho }_{f}=$ 1.225 ${\text{kg}}/{\text{m}}^{3}$ vs. ${\rho }_{p}$ = 1000 ${\text{kg}}/{\text{m}}^{3}$); hence, the virtual mass, Basset, and pressure-gradient forces can be neglected. The Faxen force comes into play when the sizes of the domains of the particle and fluid are in the same order; while in this study, the size of the particle domain is four orders of magnitude smaller than the size of the fluid domain (Chen et al. 2000). Saffman’s lift and Brownian forces are considered when the particles are sub-micrometer in size (Ounis et al. 1991; Schwarzkopf et al. 2011), while monodispersed particles with a diameter of 5 µm used in this study.

The gravitational, $\vec{F}_{g}$, and drag, $\vec{F}_{D}$, forces are given by:

9 $$\vec{F}_{g} = m_{p} \frac{{\vec{g}\left( {\rho_{p} - \rho_{f} } \right)}}{{\rho_{p} }}$$

10 $$\vec{F}_{D} = m_{p} \frac{{\left( {\vec{u} - \vec{u}_{p} } \right)}}{{\tau_{r} }}$$

where $\overrightarrow{g}$, ${\rho }_{p}$, ${\overrightarrow{u}}_{p}$ and $\overrightarrow{u}$ are the gravitational acceleration, density of the particle, velocity of the particle, and the velocity of the fluid, respectively. ${\tau }_{p}$ is the particle relaxation time defined by:

11 $$\tau_{p} = \frac{{\rho_{p} d_{p}^{2} }}{18\mu }\frac{24}{{C_{d} {\text{Re}}_{p} }}$$

where ${d}_{p}$, $\mu$, ${C}_{d}$, and ${\mathrm{Re}}_{p}$ are the diameter of the particle, the dynamic viscosity of the fluid, the drag coefficient, and the particle Reynolds number, respectively. In this study, it is assumed that the particles are spherical. For a sphere particle with a smooth surface, the drag coefficient can be estimated by Morsi et al. (1972):

12 $$C_{d} = a_{1} + \frac{{a_{2} }}{{{\text{Re}}_{p} }} + \frac{{a_{3} }}{{{\text{Re}}_{p}^{2} }}$$

where the terms ${a}_{1}$, ${a}_{2}$, and ${a}_{3}$ are the constant numbers that were estimated empirically over different ranges of ${\mathrm{Re}}_{p}$ by Morsi et al. (1972) as defined as follows:

13 $$a_{1} = 0; a_{2} = 24; a_{3} = 0\quad 0 < {\text{Re}}_{p} < 0.1$$

14 $$a_{1} = 3.69; a_{2} = 22.73; a_{3} = 0.0903\quad 0.1 < {\text{Re}}_{p} < 1$$

15 $$a_{1} = 1.222; a_{2} = 29.1667; a_{3} = - 3.8889\quad 1 < {\text{Re}}_{p} < 10$$

16 $$a_{1} = 0.6167; a_{2} = 46.5; a_{3} = - 116.67\quad 10 < {\text{Re}}_{p} < 100$$

17 $$a_{1} = 0.3644; a_{2} = 98.33; a_{3} = - 2778\quad 100 < {\text{Re}}_{p} < 1000$$

18 $$a_{1} = 0.357; a_{2} = 148.62; a_{3} = - 47500\quad 1000 < {\text{Re}}_{p} < 5000$$

19 $$a_{1} = 0.46; a_{2} = - 490.546; a_{3} = 578700\quad 5000 < {\text{Re}}_{p} < 10000$$

20 $$a_{1} = 0.5191; a_{2} = - 1662.5; a_{3} = 5416700\quad 10000 < {\text{Re}}_{p} < 50000$$

#### Boundary conditions

For modelling the airflow in geometry G1, a pressure inlet boundary condition was applied to the inlet (the hemisphere dome, see, Fig. 3b) with zero gauge-pressure (atmospheric pressure), and a mass flow outlet boundary condition was implemented to the outlet. A no-slip condition was applied to the wall. No particle tracking model was used in G1. For modelling the airflow in geometry G2, the mass flow inlet boundary condition was applied to the inlet and pressure outlet condition that was implemented on the outlet boundary with atmospheric pressure. A uniform velocity profile was used at the inlet due to the negligible effect of the fully developed inlet flow on the particle deposition in the NC (Tian et al. 2008). A no-slip boundary condition was applied to the walls. Regarding the particle phase, the inlet and outlet boundaries were set as 'Escape' and a 'Trap' boundary condition was applied to the wall to stimulate particle deposition. Trap wall condition is used to simulate the deposition of drug particles on the mucosa in the nasal cavity. 12,000 inert particles were released with zero initial velocity in the nostril through the inlet boundary.

### Validation of CFD model and mesh independence test

For validation purposes, pressure at different locations in the NC was used using geometry G1. Pressure values were recorded at different points along the NC wall. The location of the points was selected based on the available experimental data and CFD results reported in a recent study by Van Strien et al. (2021). Three different flow rates of *Q* = 10 L/min, *Q* = 15 L/min, and *Q* = 30 L/min were applied to the outlet as a mass flow outlet boundary condition. A laminar solver was used for *Q* = 10 L/min (${\mathrm{Re}}_{\mathrm{in},\mathrm{right}}=$ 823, ${\mathrm{Re}}_{\mathrm{in},\mathrm{left}}$ = 359) and *Q* = 15 L/min (${\mathrm{Re}}_{\mathrm{in},\mathrm{right}}=$ 1234, ${\mathrm{Re}}_{\mathrm{in},\mathrm{left}}$ = 585), and the SBES turbulent model was employed for *Q* = 30 L/min (${\mathrm{Re}}_{\mathrm{in},\mathrm{right}}=$ 2529, ${\mathrm{Re}}_{\mathrm{in},\mathrm{left}}$ = 1271).

**Fig. 5:**
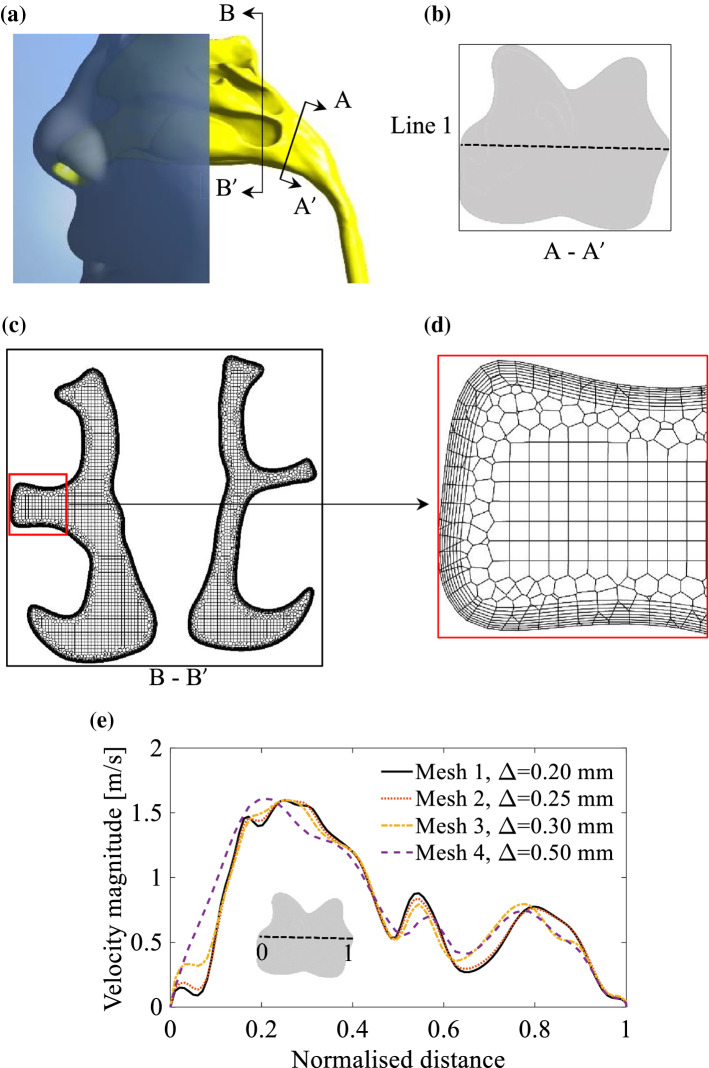
**a** Schematic of NC in G1; **b** an overview of the plane A-A’ and Line 1; **c** an overview of the meshes on the plane B-B’; **d** zoomed-in view of the mesh on plane B-B’ representing the prism layers; **e** mesh independence test based on the velocity magnitude of Line 1 when the flow rate is *Q* = 10 L/min

Initially, a mesh independence was conducted by comparing the velocity magnitude along a line in a cross-sectional plane in the nasopharynx region (see Line 1, Fig. 5b). Using the mosaic technology (introduced by ANSYS® (2020)), a poly-hexcore meshing model was used for generating the meshes. The polyhedral mesh was generated on the surface of the NC, while the interior was filled with hexahedron meshes. Polyhedral meshes were used to connect the interior hexahedrons to the inflation with eight prism layers.

For *Q* = 30 L/min, the flow is turbulent in the NC, hence, to resolve the large eddies using the LES component of the hybrid RANS-LES turbulence model (the SBES model) a sufficiently fine mesh is required. To meet this requirement, 8 prism layers were generated in the near-wall region. The size of the prism layer connected to the wall was 10% of the interior hexahedral cell length with a growth rate of 1.2. The normalised wall distance (${\text{y}}^{+}$) was less than unity on the NC wall, which satisfies the requirement of the *k-ω* SST model used in this study. Four different mesh densities of 1.1 million (Mesh 1), 3.3 million (Mesh 2), 5.1 million (Mesh 3), and 8.7 million (Mesh 4) cells at a maximum hexahedral cell length of $\Delta { = 0}{\text{.5}}$ mm, $\Delta { = 0}{\text{.3}}$ mm, $\Delta { = 0}{\text{.25}}$ mm, and $\Delta { = 0}{\text{.2}}$ mm, respectively, were generated to evaluate the mesh size. Figure 5d shows the comparison of the velocity magnitude on Line 1 obtained from different mesh configurations. The meshes with $\Delta { = 0}{\text{.2}}$ mm and $\Delta { = 0}{\text{.25}}$ mm (Mesh 1 and Mesh 2) yield almost the same distribution of the velocity magnitude. Therefore, the mesh model with $\Delta { = 0}{\text{.25}}$ mm was used in this study.

The size of time-step to resolve the eddies containing energy; hence, the Kolmogorov time scale was considered to calculate the time step size using the following equation (Landahl et al. 1989):

21 $$\tau_{n} = \sqrt {\frac{\mu }{{\rho_{f} \varepsilon }}}$$

where ${\tau }_{n}$, $\mu$, ${\rho }_{f}$, and $\varepsilon$ are the Kolmogorov time scale, fluid viscosity, fluid density, and turbulence kinetic energy per unit mass, respectively. The minimum value for Kolmogorov time scale was ${\tau }_{n}=6\times {10}^{-3}$ s. However, a smaller time step size (${\tau }_{n}=5\times {10}^{-5}$) was used for the simulations, which can guaranty that the spatial resolution was more than sufficient to resolve the turbulent flow field. The same time step size was also used for the particle tracking simulation.

**Fig. 6:**
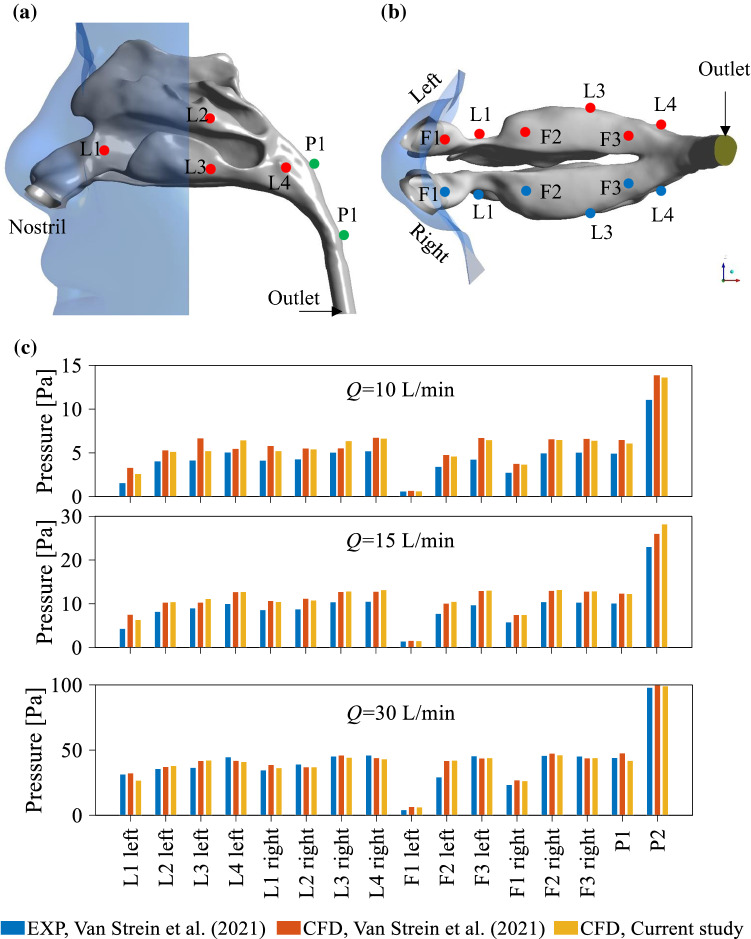
**a** An overview of different points defined on the wall of the nasal cavity used for predicting the pressure values (the model was generated through the STL file adapted from Van Strien et al. (2021) with permission); b pressure distribution on the wall of the nasal cavity obtained by the current CFD study compared with the experimental data and CFD results reported in (Van Strien et al. 2021) for a flow rate of 10 L/min, 15 L/min, and 15 L/min

To ensure that the proposed modelling technique is suitable, the flow behaviour in the G1 was modelled for different mass flow outlet rates: *Q* = 10 L/min, *Q* = 15 L/min, and *Q* = 30 L/min. The pressure values at 16 different locations on the NC wall were determined and compared with the published data. Figure 6a illustrates the location of the points on the NC wall including three points on the floor and four points on the lateral walls of both left and right sides of the NC as well as two points in the posterior region of the nasopharynx. Figure 6b represents the pressure values at the pre-defined points at different flow rates. It can be observed from this Figure that the agreement between the results obtained in this study and the results reported in the literature is reasonably good for both the laminar (*Q* = 10 L/min, *Q* = 15 L/min) and turbulent (*Q* = 30 L/min) flows.

The validation of the particle tracking model (DPM) was examined by comparing the total particle deposition efficiency (${\eta }_{T}$) in the NC predicted in this study with that reported in the literature. ${\eta }_{T}$ is defined by:

22 $$\eta_{T} = \frac{{N_{p}^{{ {\text{dep}}}} }}{{N_{p}^{ r} }} \times 100\% ,$$

where ${\text{N}}_{\text{p}}^{\text{ dep}}$ is the number of particles deposited on the wall and ${\text{N}}_{\text{p}}^{ \, {\text{r}}}$ is the number of particles released in the nostril initially. Generally, the particle transport/deposition behaviour in a fluid flow is quantified by the dimensionless Stokes number (St), which is the ratio of the particles’ momentum response time to the flow-field time scale (Krstić 2006) given by:

23 $${\text{St}} = \frac{{\rho_{p} {\text{d}}_{p}^{2} U}}{18\mu L}$$

where $U$ and $L$ are the characteristic velocity and characteristic length, normally taken as the mean flow velocity and the hydraulic diameter of the inlet (or a planar surface in the computational domain), respectively. The Stokes number is for understanding the particle transport behaviour in the fluid flow. It indicates whether the particles are in a kinetic equilibrium with the fluid phase (Tian et al. 2005). The application of the $\mathrm{St}$ depends highly on the characteristic length of the domain of interest, which changes throughout the different geometries of the NC resulting in a limitation in the calculation of Stokes number in different parts of the NC. To overcome this limitation of the $\mathrm{St}$, the inertial parameter (*IP*) was used as the criterion for the assessment of the validity of the particle phase model used in this study. The *IP* is widely used for the assessment of particle deposition in the NC and respiratory airways because the characteristic length and the characteristic velocity associated with the $\mathrm{St}$ are normalised out by using a constant flow rate. The inertial parameter is given by:

24 $$IP = Q d_{{{\text{ae}}}}^{2}$$

where ${\text{Q}}$ is the airflow rate and ${\text{d}}_{\text{ae}}$ is the equivalent aerodynamic diameter given by (Yang et al. 2012):

25 $$d_{{{\text{ae}}}} = d_{e} \sqrt {\frac{{\rho_{p} }}{1000X}}$$

where ${\text{d}}_{\text{ae}}$ is the aerodynamic diameter is defined as “the diameter of the spherical particle with a density of 1000 kg/m^3^ that has the same settling velocity as the particle under study” (Yang et al. 2012). The ${\text{d}}_{\text{e}}$ and ${\text{X}}$ are the equivalent volume diameter and the shape factor of the particle, respectively (Yang et al. 2012). For a spherical particle, the ${\text{d}}_{\text{e}}$ equals to the particle diameter and ${\text{X}}$ is unity (Yang et al. 2012). In this study, the spherical particles are assumed with a density of ${\rho }_{\text{p}}={1000} {\text{kg}}/{\text{m}}^{3}$; hence, the equivalent aerodynamic diameter is identical to the particle diameter. The *IP* is a convenient parameter that is normally used for comparing the effect of ${\text{d}}_{\text{p}}$ and ${\text{Q}}$ on the deposition efficiency. However, the use of a constant flow rate is a limitation of the inertial parameter, since it does not take into account the complicated shape of the geometry of the NC. Despite this limitation, the inertial parameter is normally used for the demonstration of deposition efficiency of particles, particularly where the determination of characteristic length is limited due to the geometry variation.

**Fig. 7:**
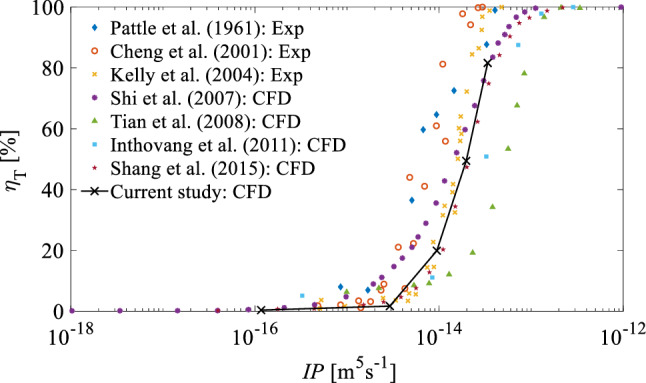
A comparison between the CFD results in the current study for total deposition efficiency (${\eta }_{T}$) as a function of the inertial parameter (${\text{IP}}$) and available data in the literature (Cheng et al. 2001; Inthavong et al. 2011; Kelly et al. 2004; Pattle 1961; Shang et al. 2015; Shi et al. 2007; Tian et al. 2008)

The effect of the inertial parameter of the total deposition efficiency in G2 was predicted using the CFD model in this study. The results were compared with the experimental and numerical data reported in the literature (Cheng et al. 2001; Kelly et al. 2004; Pattle 1961; Shang et al. 2015; Huawei Shi et al. 2007; Tian et al. 2008). Figure 7 shows that the trend of the deposition efficiency predicted in this study is similar to that of the previous studies, which implies the validity of the particle tracking model of this study. The variation in the deposition efficiency between the current and previous studies is rooted in the differences between the NC geometry of the current study and the previous studies.

## Results and discussion

### Effect of inlet flow parameters on the flow structure in the NC

**Fig. 8:**
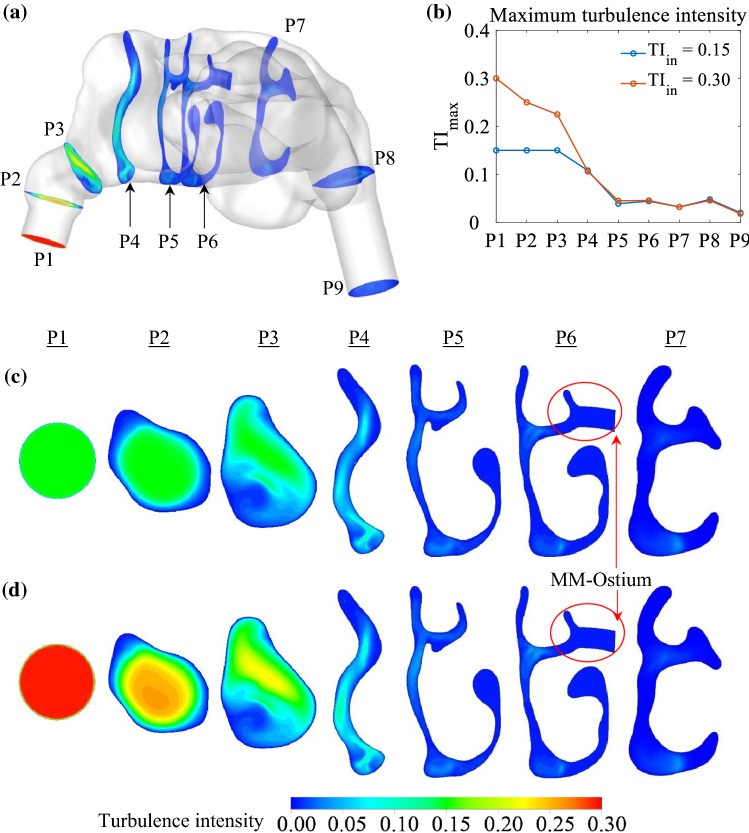
**a** Illustration of the location of different cross-sectional planes in the NC-MS combination G2); **b** maximum turbulence intensity for different cross-sectional planes; **c** instantaneous turbulence intensity (TI) contour demonstrated for different cross-sectional planes (P1–P7) in the NC-MS combination when the inlet turbulence intensity is TIin = 0.15; and **d** TIin = 0.3. The inlet mass flow rate was Q = 7 L/min

To investigate the effect of inlet flow parameters on the flow structure, several cross-sectional planes across the NC were created in critical locations as presented in Fig. 8a. Figure 8b shows the comparison of the maximum turbulence intensity (${\text{TI}}_{\text{max}})$ between the cases with ${\text{TI}}_{\text{in}}\text{=0.15}$ and ${\text{TI}}_{\text{in}}\text{=0.3}$ on planes P1-P9 for the inlet flow rate of Q = 7 L/min. Although the flow at 7 L/min is laminar in nature, an artificial turbulence intensity was added to the inlet in the Fluent software, which is hypothesised to be applied to the inlet flow in the real world using a vibrating blade at the nostril. The instantaneous contours of turbulence intensity on planes P1-P7 across the NC are presented in Fig. 8c, d. From Fig. 8b, it can be seen that the maximum turbulence intensity decreased remarkably on planes P1–P4 for both cases. However, from P5-P9 the ${\text{TI}}_{\text{max}}$ remains relatively constant. For example, in a case with ${\text{TI}}_{\text{in}}\text{=0.3}$, the maximum turbulence intensity is reduced to $\text{TI=0.045}$ in P5. This means that NC operates similar to a settling chamber transforming a turbulent flow to near laminar flow. By comparing the contours of turbulence intensity on plane P6 between the cases with ${\text{TI}}_{\text{in}}\text{=0.15}$ and ${\text{TI}}_{\text{in}}\text{=0.3}$, it can be seen that in both cases the turbulence intensities in the ostium and the MM-Ostium region are negligible (${\text{TI}}\cong {0}$). It can be inferred that the implementation of turbulence to the inlet flow has not had a significant effect on the airflow behaviour in the MM-Ostium region (see Fig. 8c, d).

**Fig. 9:**
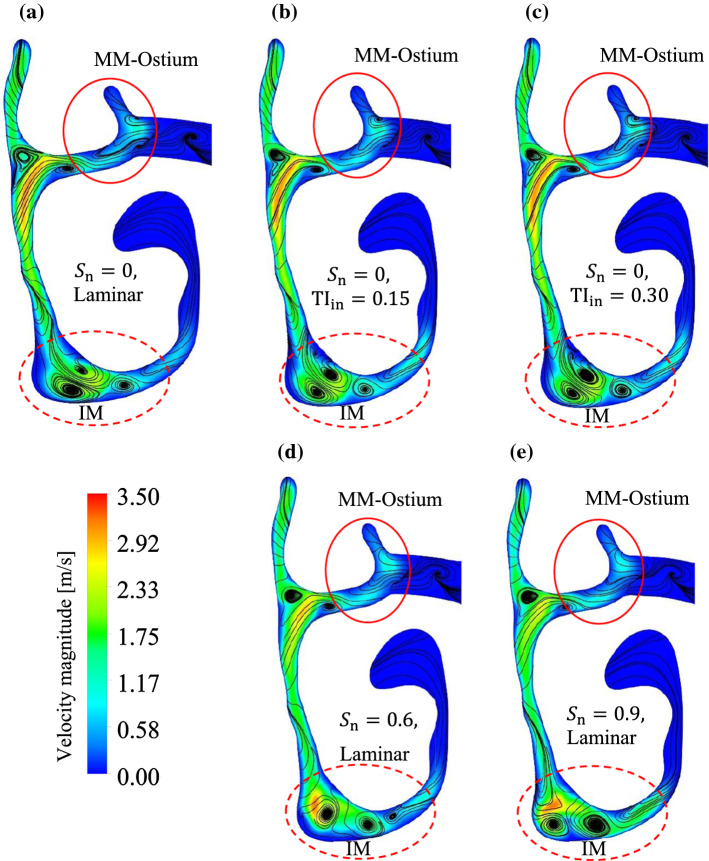
Instantaneous flow streamlines and velocity contour on plane P6 under the effect of non-swirling **a** laminar inlet flow; **b** inlet turbulence intensity of TIin = 0.15; **c** inlet turbulence intensity of TIin = 0.3; and swirling flow with **d** inlet swirl number of Sn = 0.6; **e** inlet swirl number of Sn = 0.9. Artificial turbulence was applied to the inlet for the turbulent inlet flows

Figure 9a–e presents the instantaneous streamlines and the velocity magnitude contours in plane P6 for different inlet flow parameters. As it is shown in Fig. 9a–c, in the plane P6 the flow structure after introducing turbulence at the inlet is identical to the laminar inlet flow. Based on the streamlines illustrated in Fig. 9d, e, the flow structure in the MM-Ostium region under the effect of swirling inlet flows (${S}_{\text{n}}=0.6$ and ${S}_{\text{n}}=0.9$) is also essentially identical to those of non-swirling inlet flows. Therefore, it is inferred that due to the large volume of the NC and the resistance of the nasal valve the flow stabilises and hence, the inlet flow preconditioning does not affect the flow features in the MM-Ostium region. When a swirling flow is applied to the inlet, the flow structure in the inferior meatus (marked IM in Fig. 9) undergoes some change in the number of vortices (i.e. secondary flows) due to the curvature of the anterior region. The anterior region includes the nostril and vestibule. Figure 9a–e shows that the swirling flow can influence the flow structure in the NC but mostly in the inferior meatus, where the width of the airway is wider than in the middle and superior meatuses.

**Fig. 10:**
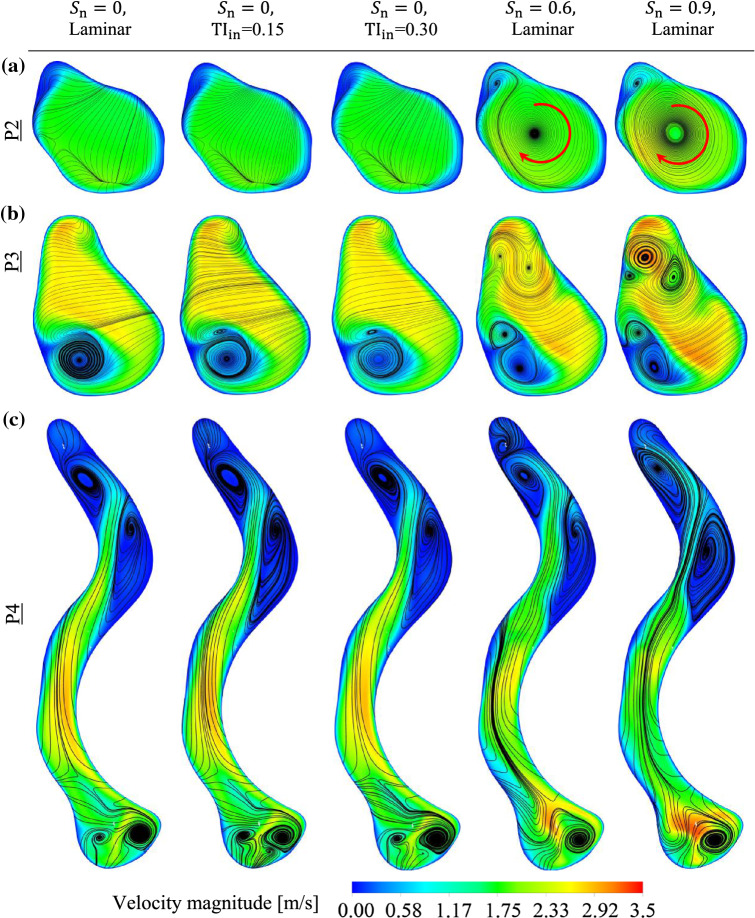
Instantaneous flow streamlines and velocity contour on **a** plane P2; **b** plane P3; and **c** plane P4 under the effect of different inlet flow parameters

To better understand the role of the nasal valve as an airway resistance, Fig. 10a–c illustrates the streamlines on planes P2–P4 for different inlet flow parameters. The vestibule is located between P2 and P3, and the nasal valve is located between P3 and P4. It is clear from this Figure that there are no significant changes in the flow streamlines in planes P2–P4 under the effect of turbulent inlet flows when compared with the laminar non-swirling inlet flow. In contrast, the flow structure in planes P2 and P3 changed significantly under the effect of swirling inlet flows. By increasing the swirl intensity of the inlet flow, the number of vortices (i.e. secondary flows) on plane P3 is increased, which demonstrates the impact of swirling flow on the flow structure in the nostril and vestibule regions. However, the influence of all inlet flow parameters on the flow structure in the plane P4 (except the IM region of P4) is negligible compared with plane P3. This implies that the constrictive nasal valve region significantly changes the flow features and reduces the effect of turbulence and swirling flow implemented to the inlet, confirming the airway resistance of the nasal valve. Hence, it might be expected that inlet flow preconditioning does not affect the efficiency of drug delivery to the MS significantly.

### Effect of inlet flow parameters on particles’ transport

Particle tracking simulation provides a detailed understanding of particle transport and deposition. To increase the efficiency of ADD to the MS, the number of particles in the MM-Ostium region should be increased and the retention of particles in that region should also be enhanced. When the ADD technique is applied, the particles are transferred to the MS through the oscillation of the air plug in the ostium. To be more specific, by the oscillating air plug during every cycle, some of the particles in the MM-Ostium region are trapped after several cycles and then, transported to the MS (Pourmehran et al. 2020). Therefore, to increase the number of particles transported to the MS through ADD, the total number of particles trapped in the oscillating air plug in several consecutive cycles should be increased, which requires longer particle retention in the MM-Ostium. To consider both the number of particles and the retention of particles in the MM-Ostium, the particles’ retention criterion (${N}_{p}^{*}$) were estimated using Eq. (2).

**Fig. 11:**
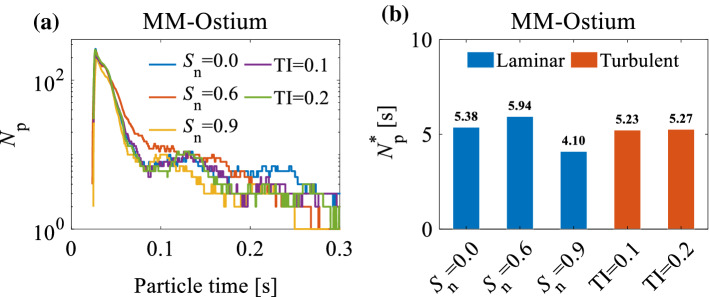
**a** The effect of inlet flow parameters on the number of particles (*Np*) in the MM-Ostium region in every time-step when *C*f = 1; **b** particle retention criterion (*Np* ∗) in the MM-Ostium region under the effect of different inlet flow parameters when *d*p = 5 μm and *C*f = 1

Figure 11a, b represents the number of particles in the MM-Ostium region, as a function of particle time, and the related ${N}_{p}^{*}$ under the effect of turbulent and swirling inlet flows when ${C}_{f}=1$ and ${\mathrm{d}}_{p}=5\upmu$m. From Fig. 11b, it can be seen that there are no significant differences in ${N}_{p}^{*}$ between the cases with turbulent inlet flows and the laminar non-swirling inlet flow in the MM-Ostium region. This behaviour originates from an identical flow behaviour between those cases (see Figs. 9 and 10).

According to Fig. 11a, b, the particle retention criterion for a swirling inlet flow with ${S}_{n}=0.6$ is greater than for a non-swirling inlet flow (${S}_{n}=0$). Therefore, the efficiency of drug delivery to the MS (through the MM-Ostium using ADD) is expected to increase when a swirling flow (i.e. ${S}_{n}=0.6$) is applied to the inlet. The differences in the ${N}_{p}^{*}$ in the cases with swirling and non-swirling flows might take place due to the variations of the flow fields in the nostril and vestibule regions (zones 1 and 2 depicted in Fig. 3e), which is discussed in the following paragraphs.

**Fig. 12:**
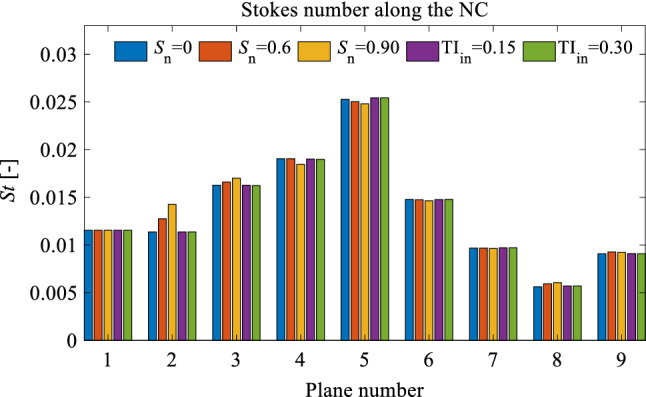
Stokes number (*St*) along the nasal cavity on planes P1-P9. The locations of P1-P9 are illustrated in Fig. 8a

Given the cross-sectional area of the nostril zone (zone 1 depicted in Fig. 3e) is almost constant, the Stokes number in this region can be calculated using Eq. (23), which yields $\mathrm{St}=0.0115$. Figure 12 presents the Stokes number on planes P1-P9 for different inlet flow parameters. It is clear from this Figure that the Stokes number along the NC is much lower than unity; hence, the particles almost follow the fluid streamlines. When a swirling flow is applied to the inlet, the airflow in the nostril swirls around the centreline of the nostril (z-axis); hence, the particles released at the nostril also swirl around the centreline of the nostril. When the particles swirl around an axis, a centrifugal force acts on the particles and drives them towards the wall. The centrifugal force has a direct relationship with the tangential velocity and particle mass.

**Fig. 13:**
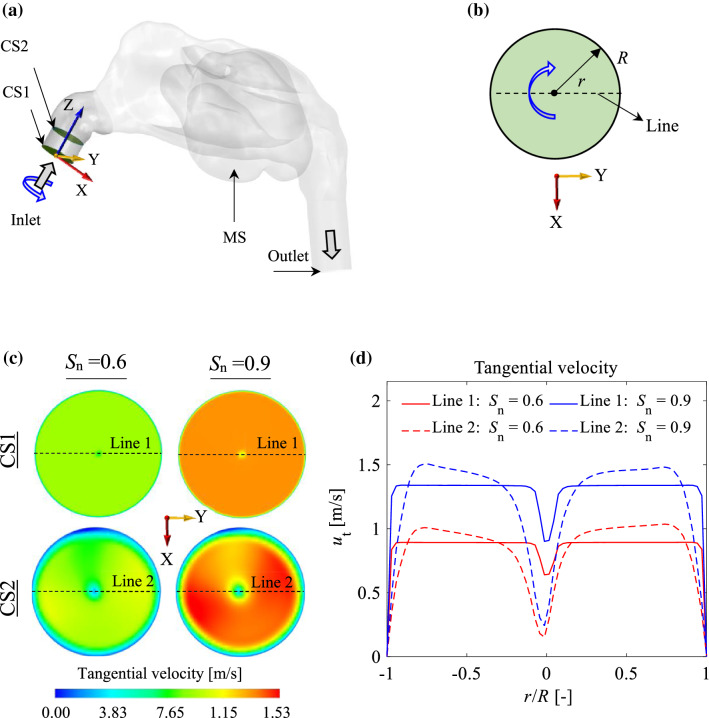
**a** An overview of NC-MS geometry (G2) representing two cross sections (CS1 and CS2) on nostril; **b** a top view of the cross section representing the rotation of the inlet flow; **c** tangential velocity contours in CS1 and CS2 for different swirl numbers (*S*_*n*_); **d** tangential velocity profiles on Lines 1 and 2 for different *S*_*n*_

**Fig. 14:**
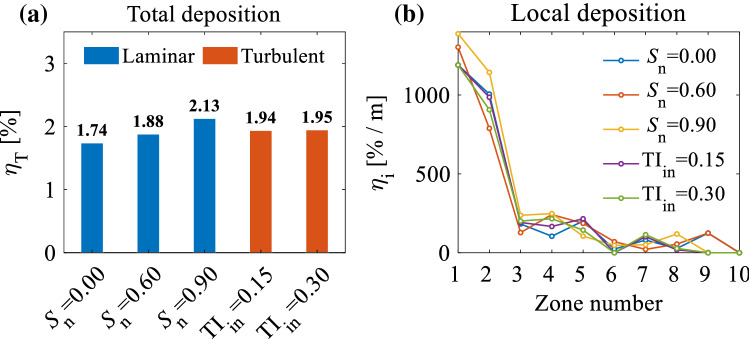
The effect of inlet flow parameters on **a** total deposition efficiency (${\eta }_{\mathrm{T}}$); and **b** local deposition efficiency per unit of area (${\eta }_{i}$)

The centrifugal force, ${F}_{c}$, can be calculated by:

26 $$F_{c} = \frac{{m_{p} u_{t}^{2} }}{r} ,$$

where ${m}_{p}$ is the particle mass, ${u}_{t}$ is the tangential velocity, and $r$ is the radial coordinate. An increase in the swirl intensity of the swirling inlet flow increases the tangential flow velocity, which increases the centrifugal force acting on the particles (see Fig. 13). Therefore, increasing the swirl number of the inlet flow increases the concentration of particles that are driven towards the wall, which can lead the particles to deposit on the wall. Figure 14a, b presents the total and local deposition efficiencies for different inlet flow parameters. This Figure shows that the swirling flow has the effect of increasing the deposition of particles on the wall, where the maximum total deposition efficiency occurs when ${\mathrm{S}}_{n}=0.9$. Figure 14b reveals that the maximum particle deposition in zones 1 and 2 (nostril and vestibule, respectively) occurs in ${\mathrm{S}}_{n}=0.9$, which implies the effect of centrifugal force on the particles in these regions, contributing to an increase in total deposition efficiency.

**Fig. 15:**
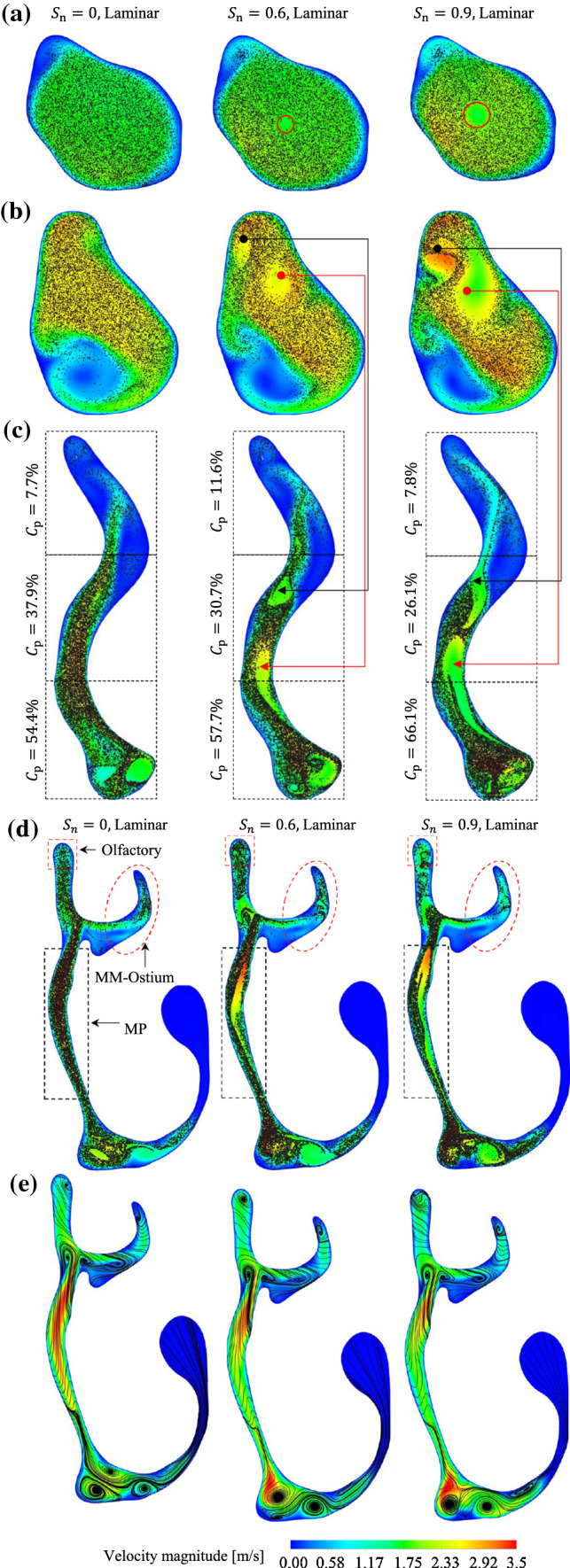
Accumulative particle distribution and instantaneous velocity contours under the effect of non-swirling and swirling flows on different planes: **a** P2, **b** P3, **c** P4, and **d** P5; **e** instantaneous flow streamlines and velocity contours on plane 5

The effect of the centrifugal force on the particles can be seen in Fig. 15a. This Figure illustrates the distribution of the particles in plane P2 for different inlet swirl numbers. According to this Figure, the application of swirling inlet flow led the particles in the central area of the nostril to move towards the wall due to the centrifugal force. Also, the central area of the nostril, which is empty of particles, becomes wider by increasing the swirl number from ${S}_{n}=0.6$–$0.9$. This implies that the centrifugal force on the particles was also enhanced when the swirl number increased. The results showed that, despite the approximately similar flow behaviour in the NC-MS geometry on planes P4–P9 (i.e. zones 4–9) for all the inlet flow preconditioning, the particle transport patterns under the effect of swirling inlet flow and non-swirling inlet flow are not identical. This could be due to the effect of flow behaviour on particle transport patterns in the first two zones (i.e. nostril and vestibule).

Figure 15a, b compares the particle distribution pattern between the non-swirling and swirling inlet flows in planes P2 and P3. The particle distribution patterns in planes P2 and P3 are similar to the flow streamlines in those planes (see Fig. 10a, b), where the Stokes number is very lower than unity. For swirling inlet flows, the particle distribution patterns in plane P4 (see Fig. 15c) are not similar to the flow streamlines in plane P4 (see Fig. 10c), even though the highest Stokes number in the nasal valve (the zone between P3 and P4) is much lower than unity ($\mathrm{St}=0.02$). The reason for the differences between the particle distribution patterns and flow streamlines in plane P4 are rooted in the particle distribution pattern formed in the upstream flow in the vestibule (zone 2). In other words, the particle distribution patterns that were formed under the effect of swirling flow in the nostril (zone 1) and vestibule regions were partially extended to the nasal valve.

Comparing the particle distribution patterns illustrated in Fig. 15b, c, it can be seen that the areas in plane P3 that are empty of particles, due to the swirling flow, were extended to plane P4. Figure 15d shows that for the cases with swirling inlet flow there is an area in the main passage of plane P5 that is almost devoid of particles, while no swirling flow in that region is observed in the streamlines of that plane (Fig. 15e). Hence, it can be inferred that the particle transport pattern in the vestibule was extended not only to the nasal valve but also to the areas beyond the nasal valve (zone 3).

In conclusion, the swirling inlet flow undergoes many changes when it passes through the nasal valve where the swirl intensity of the inlet swirling flow is almost damped. The effect of swirling flow on the particles before entering the nasal valve forms a specific particle distribution pattern. The particle distribution pattern in the nostril and vestibule not only does not disappear in the nasal valve but also partly extends to the regions beyond the nasal valve. Accordingly, the differences in ${N}_{p}^{*}$ between the non-swirling inlet flow and swirling flows presented in Fig. 11a, b) can be explained: the concentration of particles (${C}_{p}$) in the upper part of the plane P3 for ${S}_{n}=0.9$ (${C}_{p}=7.8\%$) is lower than that of ${S}_{n}=0.6$ (${C}_{p}=11.6\%$) (see Fig. 15b). So, they contribute to a lower concentration of particles in the MM-Ostium region for ${S}_{n}=0.9$ given that the particle distribution pattern in plane P3 is partly extended to the regions beyond the nasal valve. Therefore, the particle retention criterion in the MM-Ostium region for ${S}_{n}=0.9$ is lower than for other cases, as presented in Fig. 11a, b).

### Effect of fullness coefficient on the particles’ transport pattern

**Fig. 16:**
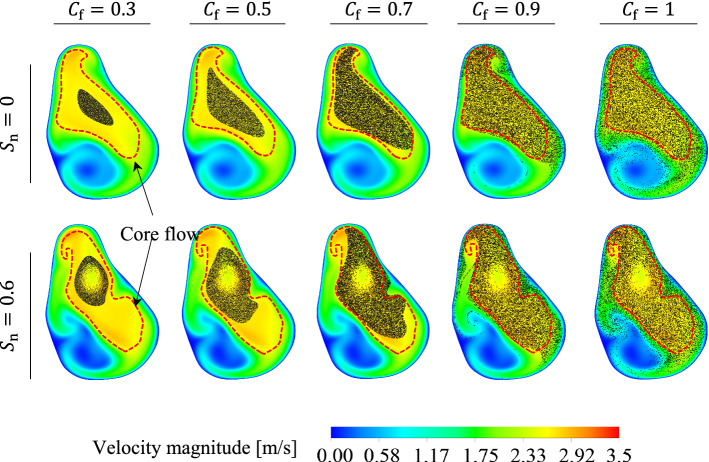
Accumulative particle distribution and instantaneous velocity contours in plane P3 for different fullness coefficient (${C}_{f}$) under the effect of non-swirling and swirling inlet flows

The effect of the fullness coefficient (${C}_{f}$) on the particle transport/deposition pattern in the NC-MS combination (G2) was investigated under the effect of non-swirling (${S}_{n}=0$) and swirling (${S}_{n}=0.6$) inlet flows. Figure 16 illustrates an overview of the accumulative particle distribution in plane P3 for different swirling numbers and ${C}_{f}$. According to this Figure, when ${C}_{f}$ decreases the concentration of particles in the core flow increases. In this study, the core flow is defined as the part of the flow where the velocity magnitude and the velocity gradient are significantly higher and lower than other parts of the flow, respectively. The core flow regions in plane P3 for ${S}_{n}=0$ and ${S}_{n}=0.6$ are illustrated in Fig. 16.

**Fig. 17:**
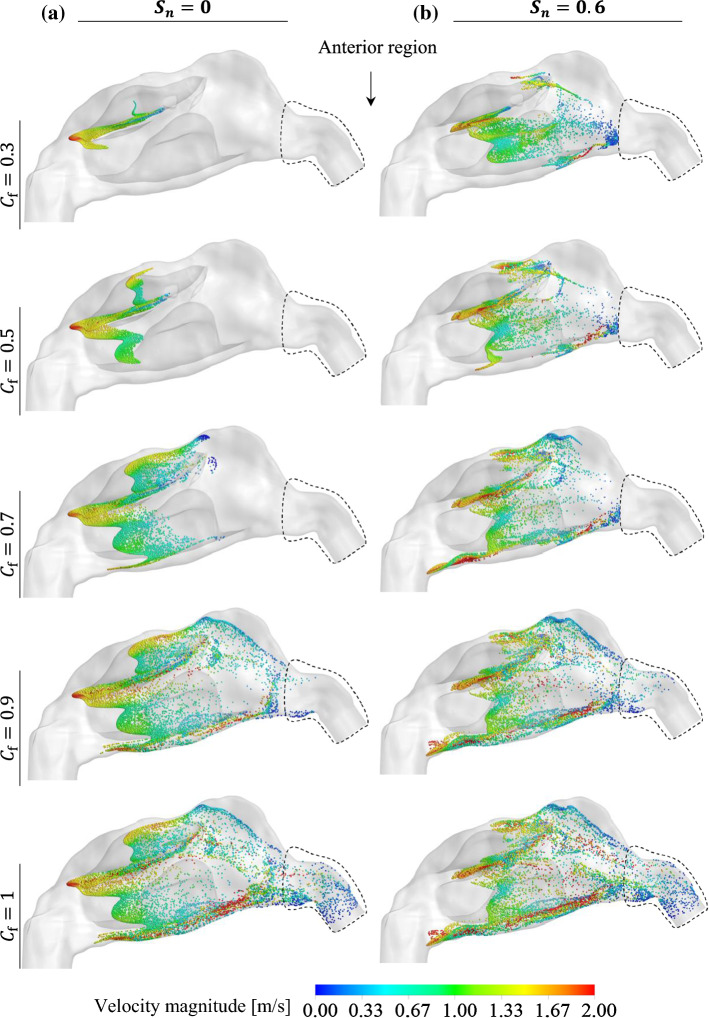
Instantaneous particle distributions in the NC for different particles’ fullness coefficient (${C}_{f}$) under the effect of **a** non-swirling inlet flow, and **b** swirling inlet flow

Given the Stokes number in this study is much lower than unity along the NC region, the particles in the NC can follow the flow. Accordingly, when the concentration of particles in the core flow region increases, the number of particles that can be transported beyond the anterior region increases. When a portion of the particles is distributed out of the core flow region, they need a longer time to be transported to the location beyond the anterior region because the velocity magnitude in that region is much lower than the core flow. To better understand the effect of ${C}_{f}$ on the particle transport pattern in the NC, Fig. 17a, b presents an instantaneous particle distribution in the NC for different fullness-coefficient. The particles were coloured, based on the particle velocity magnitude.

Figure 17a, b shows that all the injected particles were transported beyond the anterior region for ${C}_{f}\le 0.7$, demonstrating that the particles were almost transported through the core flow region. However, for ${C}_{f}=0.9$ and ${C}_{f}=1$, some particles remained in the anterior region with very low velocity, which shows these particles were not in the core flow in the anterior region (see the colour of particles in the anterior region in Fig. 17a, b). The particles that are not in the core flow region are likely to deposit on the wall due to the low velocity-magnitude, which contribute to moving the particles towards the wall and eventually depositing there.

**Fig. 18:**
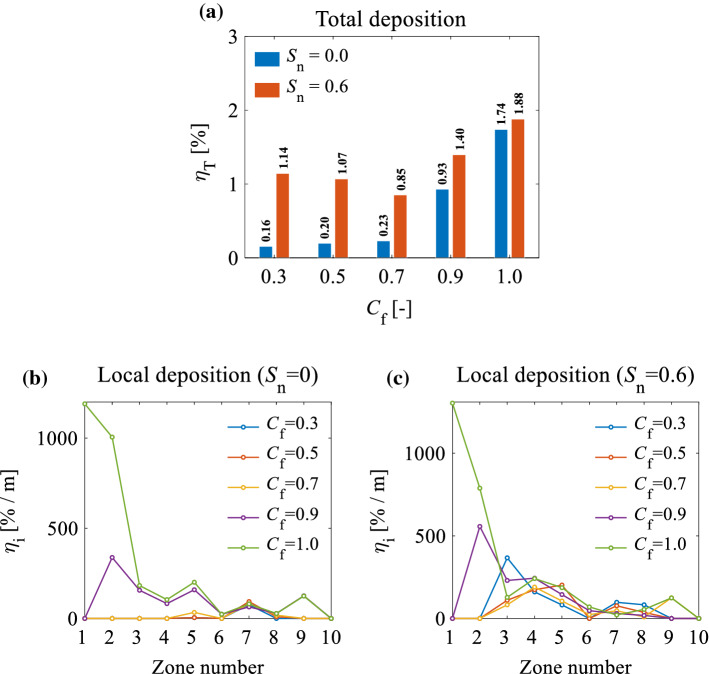
**a** The effect of fullness coefficient (${C}_{f}$) and inlet flow parameters on the total deposition efficiency (${\eta }_{T}$); **b** the effect of ${C}_{f}$ on local deposition efficiency per unit of area (${\eta }_{i}$) for a non-swirling inlet flow (${S}_{n}=0$); **c** the effect of ${C}_{f}$ on ${\eta }_{i}$ for a swirling inlet flow when ${S}_{n}=0.6$. The particle diameter of ${d}_{p}$=5 $\mu$m was used for these cases

Figure 18 presents the effect of ${C}_{\mathrm{f}}$ on the total deposition efficiency (${\eta }_{T}$) and the local deposition efficiency per unit of area (${\eta }_{i}$) for different inlet flow parameters. It can be seen from Fig. 18a that for non-swirling flows, an increase in ${C}_{f}$ increases the total deposition efficiency from an increased concentration of the particles in the region outside of the core flow. This Figure also shows that the total deposition efficiency in swirling inlet flows is higher than for non-swirling flows. This is due to the centrifugal forces acting on the particles in the core flow, which drive them towards the wall.

In continuous drug delivery, such as drug delivery using nebulisers, the deposition of the particles in the anterior region increases gradually, which can contribute to forming droplets dripping from the nostril as waste. To overcome this problem, a 70% decrease in ${C}_{f}$ is recommended for the NC-MS geometry of this study. To generalise this result, a series of different realistic NC-MS models should be analysed.

**Fig. 19:**
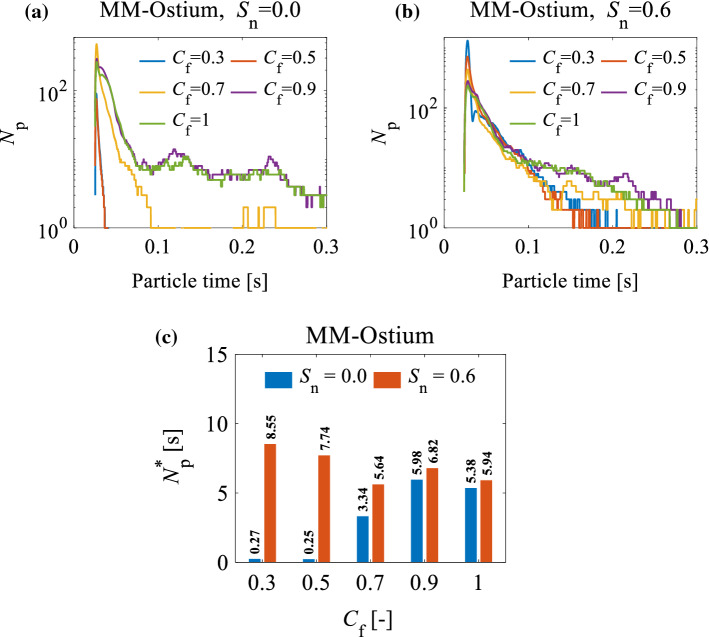
The effect of fullness coefficient (${C}_{f}$) on the number of particles that exist in the MM-Ostium region in every time-step when **a** ${S}_{n}=0$; and **b **${S}_{n}=0.6$; **c** the effect of ${C}_{f}$ on the particles’ retention criterion (${N}_{p}^{*}$) in the MM-Ostium region for non-swirling and swirling inlet flows

Figure 19a–c illustrates the number of particles in the MM-Ostium region (as a function of particle time) and the related ${N}_{p}^{*}$ under the effect of swirling and non-swirling inlet flows for different ${C}_{f}$ when ${\mathrm{d}}_{p}=5\upmu$m. It can be seen from Fig. 19c that the effect of ${C}_{f}$ on ${N}_{p}^{*}$ for swirling inlet flows is different from non-swirling inlet flows in the MM-Ostium region. This Figure shows that ${N}_{p}^{*}$ has an inverse relationship with ${C}_{f}$ when ${S}_{n}=0.6$. However, ${N}_{p}^{*}$ monotonically increases by increasing ${C}_{f}$ for non-swirling inlet flow (${S}_{n}=0$). According to Fig. 19c, the highest ${N}_{p}^{*}$ for the MM-Ostium region (${N}_{p}^{*}=8.55 \mathrm{s}$) when ${C}_{f}=0.3$ and ${\mathrm{S}}_{n}=0.6$ is approximately 45% greater than a case with ${\mathrm{S}}_{n}=0$, ${C}_{f}=1$ (a common inlet configuration). Therefore, we can infer that when ${C}_{f}=0.3$ and ${\mathrm{S}}_{n}=0.6$, the efficiency of acoustic drug delivery to the MS might be increased by up to 45% when compared with an acoustic drug delivery with a common inlet configuration. Pourmehran et al. (2021) demonstrated that using a tailored acoustic wave onto the nostril can increase the efficiency of drug delivery to the MS by more than 45-fold. Accordingly, using a swirling inlet flow (with ${\mathrm{S}}_{n}=0.6$) and fullness coefficient of ${C}_{f}=0.3$, it might be expected that the efficiency of drug delivery the MS can be increased by more than 65-fold ($1.45\times 45$) when compared with a normal inlet flow preconditioning. It should be noted that these results are valid for the NC-MS geometry (G2) of this study. A series of different realistic NC-MS models should be analysed to generalise the effect of the particles’ release diameter and inlet flow parameters on drug delivery efficiency.

## Conclusion

The main aim of the present study is to investigate the effect of inlet flow parameters and nozzle diameter (characterised by fullness coefficient) at the inlet on the airflow behaviour and drug (particle) delivery to the maxillary sinus (MS). For parametric studies, two CFD models were developed using a hybrid RANS-LES model and laminar solver and validated against the available experimental data. Artificial turbulence with intensities of ${\mathrm{TI}}_{\mathrm{in}}$ = 0.15 and ${\mathrm{TI}}_{\mathrm{in}}$ = 0.3, as well as two swirl intensities with swirl numbers of ${S}_{n} =$ 0.6 and ${S}_{n} =$ 0.9, were applied at the inlet flow with a flow rate of 7 L/min. The effect of these parameters, as well as the effect of fullness coefficients of ${C}_{f}$=0.3, 0.5, 0.7, 0.9, and 1 on the flow behaviour, particle retention criterion (${N}_{p}^{*}$), and particle deposition in the MM-Ostium region, were investigated. The results were compared with a laminar non-swirling inlet flow for ${C}_{f} =$ 1. An increase in particle retention criterion in the MM-Ostium region was calculated to quantify the increase in drug delivery to the MS region. The significant findings emerging from this study are as follows:

- The turbulence and swirl applied to the inlet flow were significantly damped when the flow passed through the nasal cavity. This implies that the nasal valve plays the role of airway resistance.
- A turbulent inlet flow has a negligible effect on particle deposition in the NC, and drug delivery to the MM-Ostium regions is negligible. However, the deposition of particles increases with an increase in the swirl intensity of the inlet flow, which comes from the increasing effect of centrifugal force acting on the particles in a swirling flow.
- The drug delivery (quantified by ${N}_{p}^{*}$) to the MM-Ostium region increases by using a swirling inlet flow at a moderate swirl number, i.e. ${S}_{n} =$ 0.6. It was also found that the variation of the fullness coefficient significantly affects the drug delivery to the MM-Ostium region. ${N}_{p}^{*}$ in the MM-Ostium region increases with a decrease in the fullness coefficient.
- In continuous drug delivery, such as drug delivery using nebulisers, the deposition of the particles in the anterior region increases gradually, which can contribute to the formation of droplets dripping from the nostril as waste. To overcome this problem, a decrease in ${C}_{f}$ is recommended.

## Limitations

The outcome of the current study is subject to the following limitation: air humidity in the nasal cavity was neglected, nebulised droplets were considered as inert particles, and an isothermal condition was assumed. Also, the particles were released at the initial time step while they were monodispersed. Moreover, zero initial velocity was used for injecting particles into the nostril, which allowed to isolate the results from the particles initial velocity; this assumption was adopted since the initial velocity of particles injected into the nostril is different for various nebulisers.

## List of symbols

$$C_{d}$$: Drag coefficient [−]
$$C_{f}$$: Fullness coefficient ($$D_{N} /D_{{{\text{in}}}}$$) [−]
$C_{p}$: Concentration of particles [%]
${\text{d}}_{{{\text{ae}}}}$: Equivalent aerodynamic diameter [m]
$$d_{p}$$: Particle diameter [m]
$D_{{{\text{in}}}}$: Diameter of inlet (nostril) [m]
$$D_{N}$$: Nozzle diameter [m]
$\Delta$: Maximum length of hexahedral cell [m]
$\varepsilon$: Turbulence kinetic energy per unit mass [m^2^s^−^^2^]
$\eta_{i}$: Local deposition efficiency per unit of area [%/m]
$\eta_{T}$: Total deposition efficiency [%]
$f$: Frequency of breathing [Hz]
$$F_{C}$$: Centrifugal force [N]
$$F_{g}$$: Gravitational force [N]
$$F_{D}$$: Drag force [N]
$$f_{s}$$: Shielding function
$$g$$: Gravitational acceleration [m/s^2^]
${\text{IP}}$: Inertial parameter [µm^2^ cm^2^/s]
*k*: Turbulent kinetic energy [m^2^/s^2^]
$$L$$: Characteristic length [m]
$m_{p}$: Particle mass [kg]
$\mu$: Dynamic viscosity of fluid [kg/m.s]
$N_{p}^{{ {\text{dep}}}}$: Number of deposited particles [−]
$N_{p}$: Number of particles [−]
$N_{p}^{ r}$: Number of particles released at the inlet [−]
$N_{p}^{*}$: Particles’ retention criterion [s]
$\rho_{f}$: Density of fluid [kg/m^3^]
$\rho_{p}$: Density of particle [kg/m^3^]
*ω*: Specific dissipation rate [1/s]
$R$: Radius of inlet (nostril) [m]
$$S_{m}$$: Source term
$$St$$: Stokes number [−]
$$S_{n}$$: Swirl number [−]
$\tau_{{\text{ij, LES}}}$: Reynolds stress tensor
$\tau_{{\text{ij, RANS}}}$: Subgrid-scale stress tensor
$\tau_{n}$: Kolmogorov time scale [s]
$\tau_{p}$: Particle relaxation time [s]
${\text{TI}}_{{{\text{in}}}}$: Inlet turbulence intensity [−]
${\text{TI}}_{{{\text{max}}}}$: Max turbulence intensity [−]
$U$: Mean fluid velocity [m/s]
$$u$$: Fluid velocity [m/s]
$$u_{a}$$: Axial velocity [m/s]
$$u_{p}$$: Particle velocity [m/s]
$$u_{t}$$: Tangential velocity [m/s]
*W*: Womersley number [−]
${\text{y}}^{ + }$: Normalised wall distance [−]

## Abbreviations

ADD: Acoustic drug delivery
CAD: Computer-aided design
CFD: Computational fluid dynamics
LES: Large-eddy simulation
MM: Middle meatus
MS: Maxillary sinus
NC: Nasal cavity
RANS: Reynolds-averaged Navier–stokes
SBES: Stress-blended eddy simulation
SST STL: Shear stress transport stereolithography
WALE: Wall-adapting local eddy-viscosity
